# Boosting algorithm improves the accuracy of juvenile forensic dental age estimation in southern China population

**DOI:** 10.1038/s41598-022-20034-9

**Published:** 2022-09-19

**Authors:** Weijie Shan, Yunshu Sun, Leyan Hu, Jie Qiu, Miao Huo, Zikang Zhang, Yuting Lei, Qianling Chen, Yan Zhang, Xia Yue

**Affiliations:** 1 School of Public Health, Southern Medical University, Guangzhou, 510515 China; 2 School of Forensic Medicine, Southern Medical University, Guangzhou, 510515 China; 3 Department of Oral Implantology, Nanfang Hospital, Southern Medical University, Guangzhou, 510515 China

**Keywords:** Dental anthropology, Forensic dentistry

## Abstract

Age estimation based on the mineralized morphology of teeth is one of the important elements of forensic anthropology. To explore the most suitable age estimation protocol for adolescents in the South China population, 1477 panoramic radiograph images of people aged 2–18 years in the South were collected and staged by the Demirjian mineralization staging method. The dental ages were estimated using the parameters of the Demirjian and Willems. Mathematical optimization and machine learning optimization were also performed in the data processing process in an attempt to obtain a more accurate model. The results show that the Willems method was more accurate in the dental age estimation of the southern China population and the model can be further optimized by reassigning the model through a nonintercept regression method. The machine learning model presented excellent results in terms of the efficacy comparison results with the traditional mathematical model, and the machine learning model under the boosting framework, such as gradient boosting decision tree (GBDT), significantly reduced the error in dental age estimation compared to the traditional mathematical method. This machine learning processing method based on traditional estimation data can effectively reduce the error of dental age estimation while saving arithmetic power. This study demonstrates the effectiveness of the GBDT algorithm in optimizing forensic age estimation models and provides a reference for other regions to use this parameter for age estimation model establishment, and the lightweight nature of machine learning offers the possibility of widespread forensic anthropological age estimation.

## Introduction

Forensic age estimation provides important evidence for identifying and enforcing age-related laws such as criminal responsibility identification, immigration, and child adoption. With the adjustment of laws in some countries, the age of criminal responsibility has been lowered. Under the Criminal Law of the People’s Republic of China, the minimum age of criminal responsibility has been decreased to 12 years old, and these revisions have led to an increase in the demand for low-age identification and higher requirements for its accuracy.

Age estimation based on the morphological process of mineralization of permanent teeth is considered a reliable means of age identification because of its highly stable mineralized morphological development, which is less susceptible to genetic^1,2^ and environmental influences, as well as the fact that teeth can be preserved longer than the average bone after death^3^. Various dental age estimation methods have been developed and widely practised in different populations, among which the Demirjian method^4^ and the Willems method^5^ are the most popular. The Demirjian method divides the development of the 7 permanent teeth in the left lower jaw into 8 stages (A–H). The stages of each tooth are converted into a specific score, and the sum of the scores for all teeth represents the maturity score. Then, the maturity score is transformed into a dental age based on the conversion tables^4^. The Willems method simplifies the steps of the Demirjian method^5^. The tooth development stage is divided into 8 stages according to Demirjian’s definition of the morphological development stage. Each stage is assigned a new score, and the sum of the scores of the 7 teeth directly represents the estimated dental age value. In essence, the Willems method is a reconstruction of the Demirjian method, which adjusts the development model parameters of the Demirjian method by reregression. Currently, there are more studies on dental age estimation in northern China. Han MQ et al. evaluated the accuracy of the Demirjian, Willems and Nolla methods for dental age estimation in adolescents aged 5 to 14 years in northern China in 2020 and obtained the highest accuracy for the Nolla method^6^. Shi et al. compared the accuracy of the Demirjian method and Willems method in estimating the dental age of Tibetan children and adolescents aged 4 to 15 years and modified the parameters of the Demirjian method in 2022 and found that the modified method is more suitable for the Tibetan population than the Demirjian and Willems methods^7^. Kwon et al. used the Demirjian method and Willems methods to determine the effect of environmental factors on dental development based on Shanghai children aged 8–14 years in 2022^8^. However, there is a lack of studies on dental age estimation for the population in southern China. Age estimation in South China still relies on bone morphology, such as the knee^9^, clavicle^10^ and carpal bone.

Currently, machine learning is widely used in medical and forensic fields, such as medical image recognition^11^ and sex estimation^12^. Fu GS et al. used seven machine learning classification algorithms, binary logistic (BLR), probit (PR) and cumulative probit (CPR) regression, linear (LDA) and quadratic (QDA) discriminant analysis, artificial neural networks (ANN) and naïve Bayes classification (NBC), to estimate sex, and LDA had the highest accuracy rate among them. Machine learning provides a considerable boost to age estimation^13^, and as a fundamental artificial intelligence algorithm, machine learning not only enables more accurate age estimation but also makes age estimation more efficient^14^. Additionally, machine learning can be constantly modified to iterate on the new system, which also brings the possibility of applying a large quantity of data that cannot be processed manually. With sufficient samples, machine learning can bring more accurate results and reduce the error of human involvement. Our study explores the appropriateness of traditional estimation methods for southern China populations while also selecting several of the most common and applicable machine learning algorithms to explore their efficiency when applied to the same data.

## Materials and methods

### Sample collection and screening

This study was approved by the Ethics Committee of Nanfang Hospital, Southern Medical University (NFEC-2022-298), and we confirm that all methods were performed in accordance with the relevant guidelines and regulations. This study was a retrospective experiment. A total of 1,477 panoramic radiograph images were collected from the Department of Stomatology, Nanfang Hospital, Southern Medical University, including 644 male samples and 833 female samples. The age of the sample ranged from 2.00 to 17.99 years old. According to the chronological age, the samples of different sexes were divided into 15 groups with a distance of 1 year (Table 1). All images were taken for diagnosis and treatment. The machine that took the panoramic radiographs was the Kodak 8000-8000C X-ray machine. Samples were collected and stored in DICOM format.

**Table 1:** Sample distribution.

Age group (years)	Female	Male	Both
2.00–2.99	5	1	6
3.00–3.99	19	11	30
4.00–4.99	33	39	72
5.00–5.99	49	45	94
6.00–6.99	59	52	111
7.00–7.99	53	43	96
8.00–8.99	41	38	79
9.00–9.99	37	34	71
10.00–10.99	37	38	75
11.00–11.99	64	51	115
12.00–12.99	123	94	217
13.00–13.99	111	73	184
14.00–14.99	60	40	100
15.00–15.99	69	42	111
16.00–16.99	36	28	64
17.00–17.99	37	15	52
Total	833	644	1477

Panoramic radiographs that were clear enough to evaluate the tooth mineralized morphology were included in the study. The sample was excluded when the patient had (1) orthodontic history or was in orthodontics, (2) multiple mandibular teeth missing more than three, (3) extra teeth appeared, (4) mandibular disease, (5) systemic diseases, and (6) history of medication affecting tooth development.

### Morphological classification

Before morphological grading, the patient's name, sex, age, and other information were hidden, and a randomly generated ID that matched the patient information was used as the only identification index. The birth date and shooting date of the patient were automatically extracted in the system, and the chronological age data of the patient were generated.

Qualified medical practitioners staged panoramic radiographs without sex and age information randomly according to the Demirjian method. All the mandibular teeth in the panoramic radiograph were evaluated, and in the final grading results, the tooth stage data of the left mandible were used. A week later, the evaluators re-evaluated 10% of the panoramic radiographs to ensure that the results were consistent between the two tests. All images were evaluated in DICOM format using greyscale displays in the Forensic Science Centre of Southern Medical University.

### Machine learning methods

After several rounds of testing, the data were randomly sliced into a training set and a test set in a 3-to-1 ratio to obtain the best estimation efficiency. The training set included approximately 984 image slices, and the test set included approximately 493 images. Machine learning was performed using several simple machine learning algorithms, with age as the dependent variable and gender and Demirjian mineralization morphological staging of seven teeth as independent variables, to complete the age estimation model architecture. The following machine learning supervised regression algorithms were tested in the study: support vector regression (SVR), backpropagation neural network (BPNN), random forest, AdaBoost, K-nearest neighbour (KNN), light gradient boosting machine (Light GBM), XGBoost, extra trees, decision trees, gradient boosting decision tree (GBDT), and CatBoost. The machine learning results were evaluated using the same metrics.

All the samples were randomly divided into a training set and a test set at a ratio of 3:1 after the default (SPSSPRO, 1.1.5) randomization. The following are the specific machine learning parameters used in this study. In the decision tree, the minimum number of samples for internal node splitting was 2, the minimum number of samples for leaf nodes was 2, the minimum sample weight in the leaf nodes was 0, the maximum depth of the tree was 10, the maximum number of leaf nodes was 50, and the threshold for node division impurity was 0. In AdaBoost, the number of base classifiers was 100, and the base classifier was a decision tree classifier with a learning rate of 1. In the random forests and extra trees, the minimum number of samples for internal node splitting was 2, the minimum number of samples for the leaf nodes was 1, the minimum sample weight in the leaf nodes was 0, the maximum depth of the tree was 10, the maximum number of leaf nodes was 50, the node division impurity threshold was 0, and the number of decision trees was 100. There was put-back sampling and no out-of-bag data testing. In the GBDT, the number of base learners was 100, the learning rate was 0.1, the ratio of no put-back sampling was 1, the minimum number of samples for internal node splitting was 2, the minimum number of samples for leaf nodes was 1, the minimum sample weight in the leaf nodes was 0, the maximum depth of the tree was 10, the maximum number of leaf nodes was 50, and the node division impurity was 0. In the LightGBM, the base learners are GBDTs, the number of base learners was 100, the learning rate was 0.1, the L1 regularization term was 0, the L2 regularization term was 1, the sample levy sampling rate was 1, the tree feature sampling rate was 1, the node splitting threshold was 0, the minimum sample weight in the leaf nodes was 0, the maximum depth of the tree was 10, and the minimum number of samples in the leaf nodes was 10. In the CatBoost, the number of iterations was 100, and the learning rate was 0.1. In the KNN, the number of leaves was 30, and the number of nearest neighbours was 5. In the BPNN, the learning rate was 0.1, the L2 regular term was 1, the number of iterations was 1000, and the number of hidden layer 1 neurons was 100. In the SVR, the penalty factor was 1, the kernel function In the XG boost algorithm, the base learner was GBTree, the number of base learners was 100, the learning rate was 0.1, the L1 regular term was 0, the L2 regular term was 1, the sample sampling rate was 1, the tree feature sampling rate was 1, the node feature sampling rate was 1, the minimum weight of the samples in the leaf nodes was 0, and the maximum depth of the tree was 10. All operations were performed using SPSSPRO (1.1.5).

### Statistical analysis

The dental ages estimated by the Demirjian method and the Willems method were compared to the chronological age. A positive result designated an overestimation, and a negative result indicated an underestimation of age. The deviation between dental age and chronological age and the mean absolute error (MAE) were used as indicators of the estimation effect in this study. MAE indicates the mean of individual errors in age estimation. The lower the MAE is, the higher the age estimation reliability. *P* < 0.05, < 0.01, and < 0.001 were determined as three levels of significance. All statistical procedures and data visualization were performed using R (4.1.0) and SPSSPRO (1.1.15).

### Ethical approval

This research study was conducted retrospectively from data obtained for clinical purposes. This study was approved by the Ethics Committee of Nanfang Hospital, Southern Medical University (NFEC-2022-298).

### Approval for animal and/or human experiments

The material use was reviewed and approved by the Ethics Committee of Nanfang Hospital, Southern Medical University.

### Informed consent

Informed consent for this study was waived by the Ethics Committee of Nanfang Hospital, Southern Medical University.

## Results

### Traditional estimation methods

The intraobserver and interobserver reproducibility were satisfactory, with Kappa coefficients of 0.930 and 0.963, respectively.

**Figure 1:**
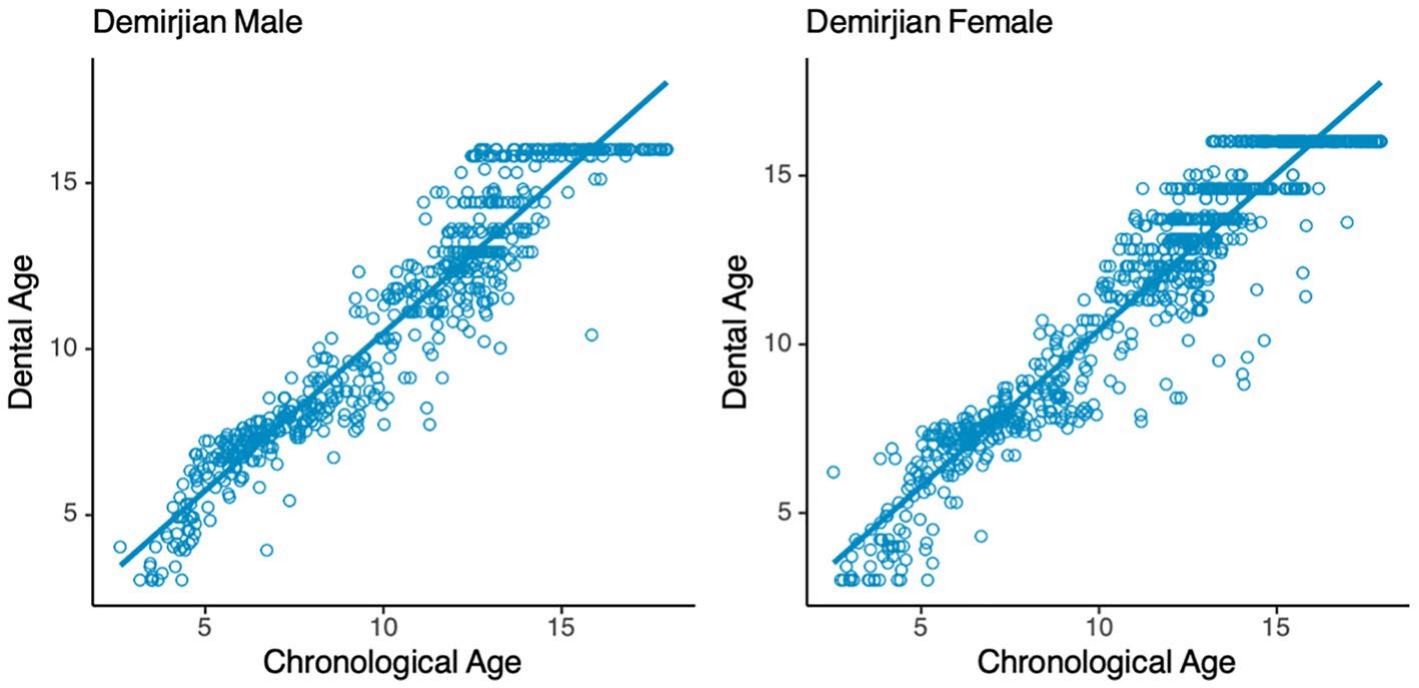
Distribution of chronological age and dental age (*Demirjian*).

**Figure 2:**
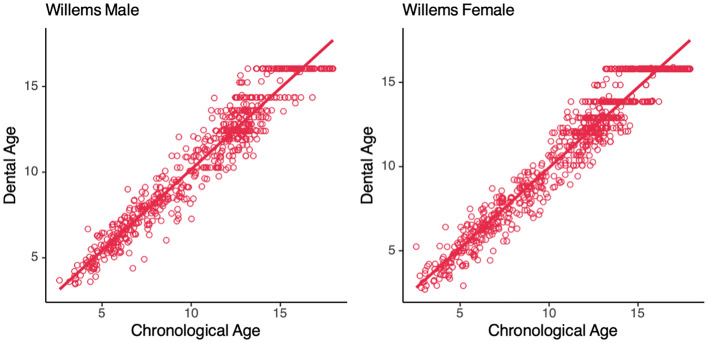
Distribution of chronological age and dental age (*Willems*).

The accuracy of the parameters of the Demirjian method with those of the Willems method in estimating the age of the South China population was compared using the same metrics. The results show that the Demirjian method significantly overestimated the age of both gender groups (Table 2), with males overestimating by 0.42 years and females by 0.31 years, and the Willems method overestimated the mean age of the male group by 0.09 years and underestimated the mean age of the female group by 0.16 years (Table 2), with the Willems method presenting results with much less error. Comparing the fit of the two to age, the results fitted by the Willems scheme also presented a better linear correlation (Figs. 1, 2). Compared to the Demirjian method, the Willems method has less error and is more suitable for age estimation in the South China population.

**Table 2:** The fitting effect of different models.

Method	Gender	CA^a^	DA^b^	AD^c^	*P* value^d^	MAE (< 18)	MAE (< 16)
Demirjian	Female	11.18(3.83)	11.49(3.72)	0.31(1.18)	0.00***	0.95	0.94
Demirjian	Male	10.68(3.76)	11.10(3.74)	0.42(1.15)	0.00***	0.96	0.96
Willems	Female	11.18(3.83)	11.02(3.78)	− 0.16(0.98)	0.00***	0.78	0.73
Willems	Male	10.68(3.76)	10.77(3.70)	0.09(0.98)	0.02***	0.77	0.76
Modified	Female	11.18(3.83)	11.16(3.73)	− 0.02(0.97)	0.61	0.77	0.71
Modified	Male	10.68(3.76)	10.68(3.55)	0.01(0.96)	0.83	0.76	0.72

*MAE* Mean absolute error.

^a^Chronological age.

^b^Dental age.

^c^Age deviation, AD = dental age–chronological age. Chronological age, dental age and the age deviation are given as the mean (standard deviation).

^d^Paired T test significance of chronological age and dental age.

Since the Willems method is more suitable for the South China population, the Willems parameters of each tooth and chronological age were fitted by linear regression with a nonintercept in each gender group to generate a model suitable for the South China population. After the Willems scores were weighted by regression coefficients, a modified model was generated (Tables 3, 4). The results of age estimation using the modified model parameters showed that the fitting effect was better than that of the Willems method. In the male group, the mean age was overestimated by 0.01 years and underestimated by 0.02 years in the female group. *No significant differences were observed between chronological age and dental age (P* > *0.05). The P value improved by one notch from* < *0.05 to* > *0.05.* However, the MAE for the male and female groups of the modified model was essentially the same as that of the Willems method. This shows that there are limited improvements to the MAE in terms of math. The modified model also adjusted MAE for each age group to the level of less than 1 year, which is more advantageous in age estimation for the large population.

**Table 3:** The tooth score of the modified model (left mandibular teeth, *male*).

Tooth	A	B	C	D	E	F	G	H
Central incisor	–	–	0.90	0.80	0.80	1.00	1.11	1.17
Lateral incisor	–	–	0.43	0.49	0.57	0.84	1.02	1.27
Canine	–	–	–	0.03	0.24	0.37	0.85	1.48
First bicuspid	0.19	0.72	0.97	1.44	1.91	2.62	3.14	3.66
Second bicuspid	0.05	0.03	0.07	0.16	0.20	0.27	0.24	0.70
First mola	–	–	–	1.07	1.77	2.49	3.03	3.34
Second molar	0.17	0.46	0.69	0.77	1.27	1.94	2.40	4.03

**Table 4:** The tooth score of the modified model (left mandibular teeth, *female*).

Tooth	A	B	C	D	E	F	G	H
Central incisor	–	–	1.86	2.23	2.38	2.87	3.25	3.20
Lateral incisor	–	–	–	0.30	0.33	0.50	0.80	0.71
Canine	–	–	0.63	0.57	0.65	1.14	1.81	2.11
First bicuspid	− 1.10	− 0.17	0.19	0.47	0.69	1.47	1.83	2.53
Second bicuspid	− 0.20	0.01	0.29	0.18	0.37	0.37	0.58	1.60
First mola	–	–	–	0.63	0.92	1.59	1.86	2.26
Second molar	0.11	0.09	0.17	0.26	0.54	1.05	1.71	3.30

### Machine learning results

Optimization of the model by simple mathematical fitting alone was limited and did not result in a significant improvement in the accuracy of the estimates. We put all the samples into machine learning, using the morphological staging and gender of the seven left mandibular teeth as independent variables and the actual age as the dependent variable, in an attempt to optimize the assessment of efficacy using machine learning.

**Figure 3:**
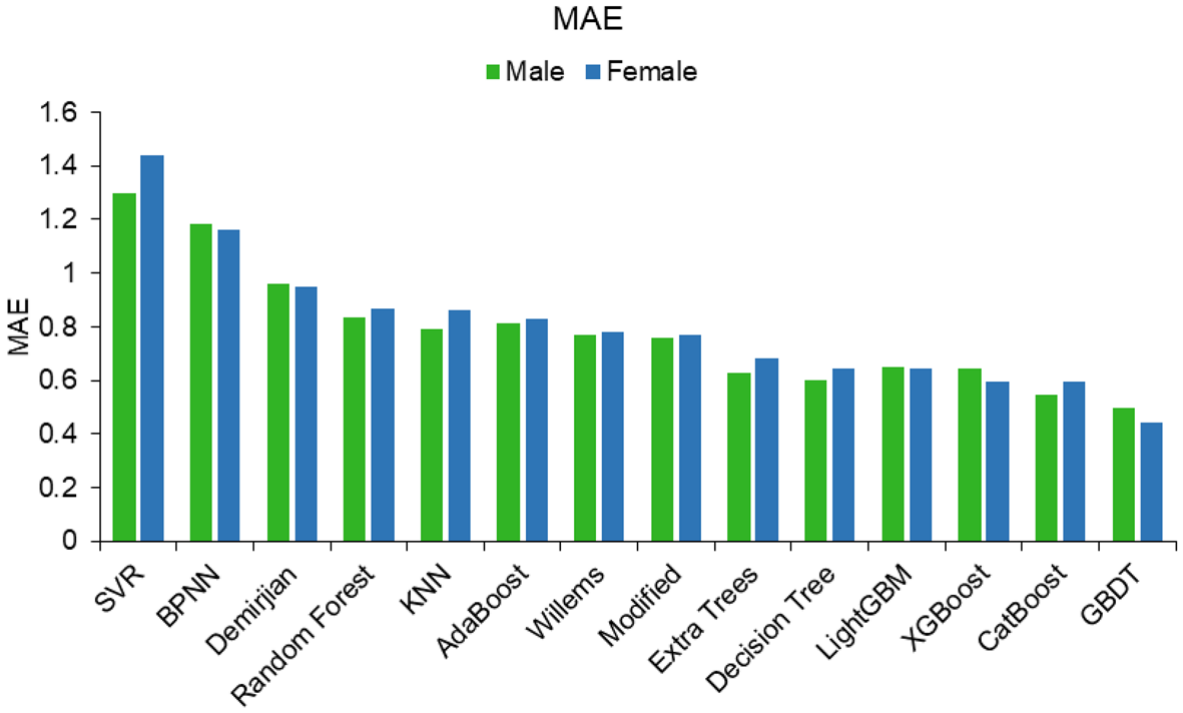
MAE comparison of different integration algorithms with traditional mathematical fitting methods.

**Figure 4:**
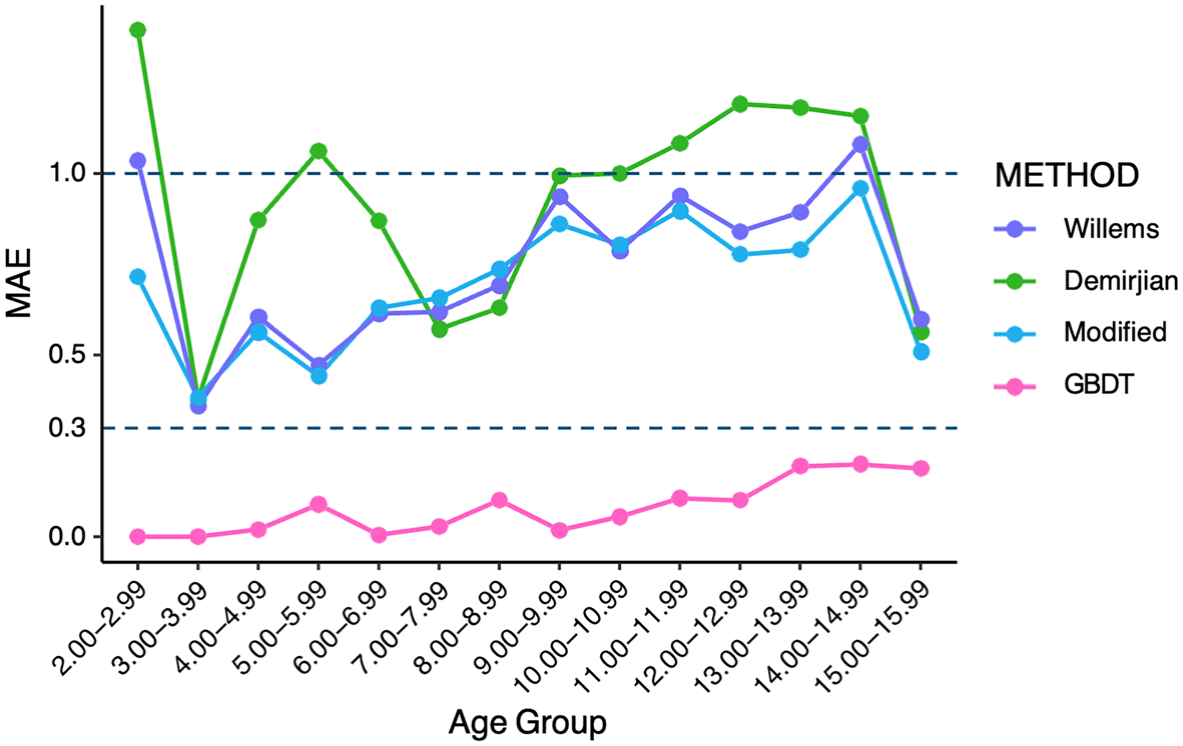
Error in estimating dental age by different methods in *male groups* (2–16 years).

**Figure 5:**
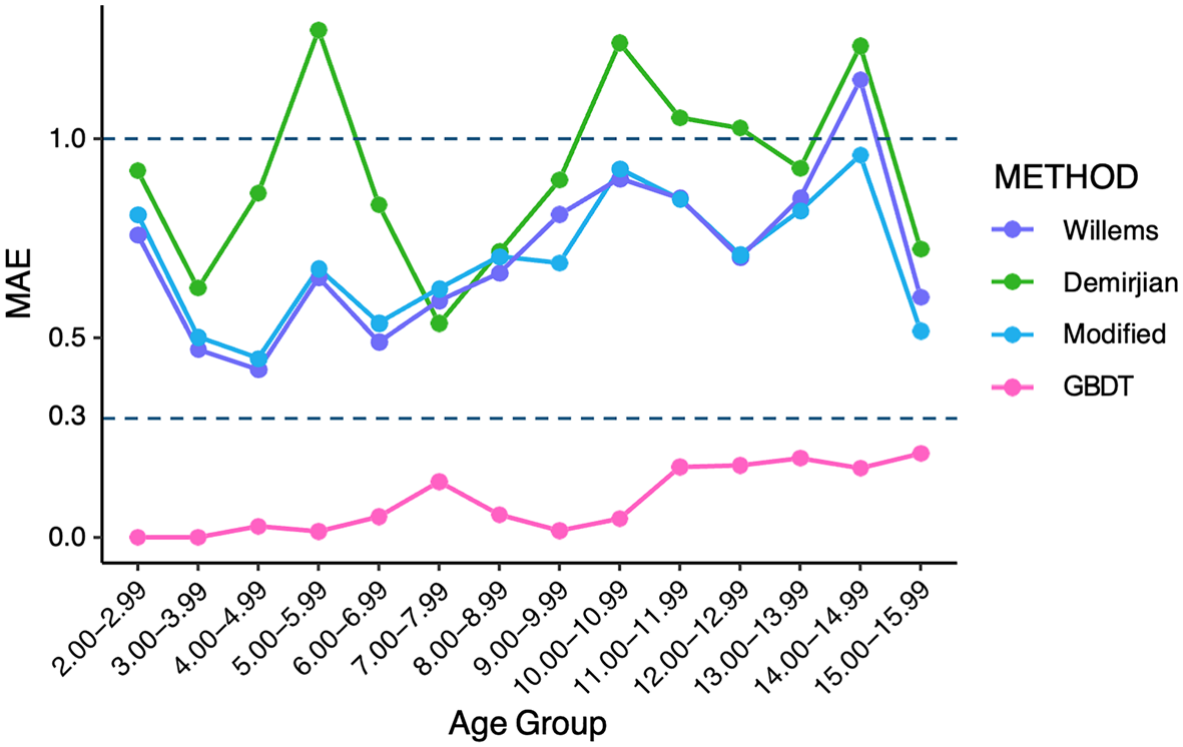
Error in estimating dental age by different methods in *female Groups* (2–16 years).

The effectiveness of each machine learning algorithm in optimizing the model was compared, and the results showed that LightGBM, XGBoost, extra -trees, decision tree, CatBoost and GBDT were effective in improving the estimation of the model, with XGBoost, CatBoost and GBDT all being algorithms under the boosting framework. The GBDT algorithm had the greatest improvement on the estimation effect (Fig. 3). After this study obtained the optimal model based on the training set using different machine learning algorithms, this study used a test set to validate the performance and evaluate the effectiveness of the model. The results of GBDT showed that the dental age estimation model achieved the expected results with an MAE of 0.523 in the training set and 0.441 in the test set for females and an MAE of 0.534 in the training set and 0.495 in the test set for males. 0.495, and the model showed the same good evaluation (Table 5). Additionally, its estimation effectiveness was optimized for each age group, with the MAE dropping below 0.25 for all age groups in the test set (Figs. 4, 5). The above results show that machine learning algorithms, particularly the boosting algorithm, can significantly improve the estimation efficiency of the model and improve the age estimation accuracy. The successful optimization shows that the dental age estimation model efficacy can be optimized by the machine learning algorithm to generate a dental age estimation model that is more suitable for a specific regional population. The optimized model can provide more accurate results for forensic age estimation and can better meet the needs of age estimation in actual forensic identification.

**Table 5:** The results of the machine learning algorithm model for females and males.

Set	MSE		RMSE		MAE		R^2^	
	Female	Male	Female	Male	Female	Male	Female	Male
**Decision tree**								
Training	0.799	0.865	0.894	0.93	0.684	0.679	0.933	0.938
Testing	0.668	0.659	0.817	0.812	0.643	0.602	0.948	0.954
**Random forests**								
Training	0.674	0.789	0.821	0.888	0.565	0.654	0.946	0.94
Testing	1.495	1.244	1.223	1.115	0.868	0.834	0.866	0.924
**AdaBoost**								
Training	1.15	1.172	1.072	1.082	0.78	0.796	0.907	0.916
Testing	1.255	1.123	1.12	1.06	0.828	0.815	0.891	0.921
**GBDT**								
Training	0.604	0.654	0.777	0.809	0.523	0.534	0.951	0.952
Testing	0.426	0.628	0.652	0.793	0.441	0.495	0.963	0.957
**Extra trees**								
Training	0.74	0.809	0.86	0.899	0.655	0.665	0.942	0.943
Testing	0.786	0.702	0.887	0.838	0.682	0.626	0.923	0.947
**CatBoost**								
Training	0.578	0.747	0.76	0.864	0.56	0.613	0.953	0.945
Testing	0.683	0.584	0.827	0.764	0.594	0.545	0.944	0.961
**KNN**								
Training	1.268	1.065	1.126	1.032	0.795	0.748	0.896	0.923
Testing	1.458	1.134	1.207	1.065	0.862	0.792	0.88	0.922
**BPNN**								
Training	2.117	2.632	1.455	1.622	1.105	1.268	0.827	0.817
Testing	2.107	2.403	1.452	1.55	1.16	1.182	0.827	0.813
**SVR**								
Training	4.096	2.816	2.024	1.678	1.659	1.29	0.677	0.798
Testing	2.945	2.794	1.716	1.672	1.437	1.3	0.727	0.8
**Light GBM**								
Training	0.76	0.801	0.872	0.895	0.634	0.67	0.937	0.943
Testing	0.676	0.839	0.822	0.916	0.642	0.648	0.947	0.938

*MSE* Mean square error, *RMSE* Root mean squared error, *MAE* Mean absolute error.

Overall, the statistical results showed that the age of the southern China population was significantly overestimated by the Demirjian method, while the Willems method overestimated the age of males and underestimated the age of females to a lesser degree. The linear regression model without an intercept based on the Willems method can better fit the age of both gender groups and has been proven to be one of the available optimization methods. The machine learning algorithm significantly improves the age prediction accuracy of the population in South China, which is more valuable for practical application (Figs. 4, 5). GBDT, as a well-known algorithm in the boosting framework, has also excelled in reducing age estimation errors.

## Discussion

The sample selection did have some bias in each group, but the broad baseline was not problematic and did not affect the study results. The total sample size and the sample size of each group were completely adequate. We believe that such a broad baseline is sufficient to characterize the developmental characteristics of a population in a region.

The Demirjian method shows a trend of overestimating chronological age in both the male and female groups of the southern China population, and the error was large, so it is considered unsuitable for the age estimation of the southern China population. This result is consistent with many studies in China^6,15,16^ or Asian regions^17^. The Demirjian method was developed for age estimation of French-Canadians children, and the overestimation of the results in and around China suggests that the results of the age estimation are probably matched by the geographical proximity of the population. The poor linear relationship between the two may be the reason why this method cannot give a good estimation result, which is fundamentally the difference between the development patterns. The Willems method had a smaller margin of error than the Demirjian method. The Willems parameter modification was also due to the Demirjian method's unsatisfactory estimation effect in Belgian children, so the overall estimation results were revised down and thus showed a corresponding better estimation performance in the population of southern China. Similarly, the results of the Willems method are similar in China^6,18^ and its neighbouring regions^19^.

The new model proposed new weights, eliminated deviations between chronological age and dental age in the total sample and showed good results in improving estimation accuracy. This suggests that the reconstruction of a linear model can effectively improve estimation accuracy when the original linear correlation is good to propose a more accurate model for the population in each region. This study also proves that morphology-based dental age estimation is an effective method for estimating the chronological age of young children and adolescents.

Optimization of the model by machine learning resulted in a significant reduction in MAE for each age group, with the GBDT algorithm performing best in the optimization. Possible reasons for this optimization include the following. (1) Machine learning eliminated the use of transformation tables, reducing the error in the model process. (2) The boosting algorithm is first trained on the full sample set, and the training produces a series of weak learners. In the next round of training, the training set is kept constant, but the weights of the correct samples are reduced, and the weights of the incorrect samples from the previous round are increased. A function is found to fit the residuals from the previous round, generating a strong learner until the residuals are sufficiently small or a set maximum number of iterations is reached to stop the iterations. As tooth development is not completely linear and teeth tend to develop faster and then slower, such a general, nonlinear estimation method may be more suitable for age assessment based on the developmental pattern of tooth mineralization.

The GBDT algorithm is flexible enough to handle various types of data, including continuous and discrete values, and takes full account of the weights of each classifier. GBDT has been shown to excel in a number of areas^14,20,21^, but our study is the first to apply GBDT to dental age estimation. Our study also demonstrates that GBDT can break the accuracy limits of traditional dental age estimation and lead to better results for forensic age estimation. The GBDT algorithm in the boosting framework can estimate the age of the population more accurately, suggesting that machine learning can effectively improve the accuracy of dental age estimation models for all age groups, and with a sufficient number of prior samples, a model with higher accuracy can be proposed for each region. Compared to traditional dental age estimation methods, the age estimation models obtained by machine learning algorithms are more easily applied in other regions and are simpler to implement. However, it is noteworthy that seemingly large enough datasets and enough learning algorithms have existed for decades, yet despite thousands of papers applying machine learning algorithms to medical data, few papers have made meaningful contributions to solving practical problems. This study proposes practical age estimation parameters to facilitate the application of dental estimation in forensic anthropology in southern China, and the conclusion that GBDT is effective in improving age estimation efficacy, as demonstrated in this study, also provides a reference for developing forensic dental age estimation in other regions.

Unlike end-to-end estimation schemes with no human intervention and automatic extraction of dental features for age estimation using CNN algorithms^22^, this study manually determines the staging and then uses machine learning algorithms to continuously iterate to obtain the most appropriate age estimation model. This estimation process can be accomplished using a lightweight computer terminal, which eliminates the tediousness of using a large server for model training. In comparison, the MAE obtained by the GBDT algorithm (< 0.5) is also lower than that of the end-to-end solution (0.83). Machine learning algorithms bring more possibilities for the wide application of age estimation in forensic science.

The samples in this study were collected from southern China hospitals, which have certain regional limitations, and no ethnographic traceability was performed to determine whether the samples had lived in southern China for generations. The boosting algorithm achieved good results in this study, but data from other regions were not collected for the trial, which needs to be corroborated by subsequent trials. In addition, the study did not achieve fully automated dental age estimation, and further research is needed to develop an end-to-end model.

We believe that the accuracy of machine learning will be further improved with the increase of sample size. The sample size of this study is limited, and the highest degree of improvement of machine learning cannot be verified, and the maximum optimization effect of different machine learning solutions has to be verified. The efficacy test of this study was conducted only in the samples collected by us, and the application effect of our proposed protocol in clinical practice needs further study.

## Conclusion

We compared the validity of traditional age assessment programs with machine learning programs for age estimation in a South China population. The results showed that the Demirjian method is more accurate than the modified and Willems methods in dental age estimation of children aged 7–8 years in South China, while the results showed that the Demirjian method is less accurate than the Willems method in other age groups, and the accuracy of age assessment can be improved by reweighting the parameters. Training the same data using machine learning methods can effectively improve the age estimation accuracy, with several algorithms in the boosting framework performing better than traditional methods, and GBDT can lead to the most accurate estimation. This boosting-based model architecture model eliminates the tedious steps of traditional models, while the lightweight machine learning model architecture can be implemented more easily across institutions. We demonstrate that schemes such as GBDT can effectively optimize forensic age estimation accuracy and can provide a reference for model architectures for age assessment in various regions.

## Data Availability

The datasets used and/or analyzed during the current study are available from the corresponding author on reasonable request.
